# Mediators of Neuropathic Pain; Focus on Spinal Microglia, CSF-1, BDNF, CCL21, TNF-α, Wnt Ligands, and Interleukin 1β

**DOI:** 10.3389/fpain.2021.698157

**Published:** 2021-08-25

**Authors:** Paul A. Boakye, Shao-Jun Tang, Peter A. Smith

**Affiliations:** ^1^Department of Anesthesiology, Renaissance School of Medicine, Stony Brook University, Stony Brook, NY, United States; ^2^Neuroscience and Mental Health Institute and Department of Pharmacology, University of Alberta, Edmonton, AB, Canada

**Keywords:** central sensitization, dorsal horn, nerve injury, neuropathy, cytokine, chemokine, growth factor, synaptic transmission

## Abstract

Intractable neuropathic pain is a frequent consequence of nerve injury or disease. When peripheral nerves are injured, damaged axons undergo Wallerian degeneration. Schwann cells, mast cells, fibroblasts, keratinocytes and epithelial cells are activated leading to the generation of an “inflammatory soup” containing cytokines, chemokines and growth factors. These primary mediators sensitize sensory nerve endings, attract macrophages, neutrophils and lymphocytes, alter gene expression, promote post-translational modification of proteins, and alter ion channel function in primary afferent neurons. This leads to increased excitability and spontaneous activity and the generation of secondary mediators including colony stimulating factor 1 (CSF-1), chemokine C-C motif ligand 21 (CCL-21), Wnt3a, and Wnt5a. Release of these mediators from primary afferent neurons alters the properties of spinal microglial cells causing them to release tertiary mediators, in many situations *via* ATP-dependent mechanisms. Tertiary mediators such as BDNF, tumor necrosis factor α (TNF-α), interleukin 1β (IL-1β), and other Wnt ligands facilitate the generation and transmission of nociceptive information by increasing excitatory glutamatergic transmission and attenuating inhibitory GABA and glycinergic transmission in the spinal dorsal horn. This review focusses on activation of microglia by secondary mediators, release of tertiary mediators from microglia and a description of their actions in the spinal dorsal horn. Attention is drawn to the substantial differences in the precise roles of various mediators in males compared to females. At least 25 different mediators have been identified but the similarity of their actions at sensory nerve endings, in the dorsal root ganglia and in the spinal cord means there is considerable redundancy in the available mechanisms. Despite this, behavioral studies show that interruption of the actions of any single mediator can relieve signs of pain in experimental animals. We draw attention this paradox. It is difficult to explain how inactivation of one mediator can relieve pain when so many parallel pathways are available.

## Introduction

This review outlines aspects of the etiology of neuropathic pain at both the spinal and peripheral level. A variety of chemical mediators effect communication between the various cell types involved in the generation of pathological pain. We focus on mediators that affect spinal microglia, mediators released from microglia and their actions on their target cell types.

Peripheral nerve trauma, post herpetic neuralgia, spinal cord injury, traumatic brain injury, stroke and neuropathies associated with chemotherapy, diabetes or HIV infection can give rise to intractable neuropathic pain (1–13). Neuropathic components also contribute to pain associated with COVID-19, multiple sclerosis, fibromyalgia, migraine, osteoarthritis, rheumatoid arthritis, autoimmune disease, and complex regional pain syndromes (14–23). Although the signs and symptoms of neuropathic pain are similar in males and females, it is now well-established that the underlying cellular mechanisms are very different (24–33). Unlike nociceptive pain, which signals and protects an individual from tissue injury, neuropathic pain persists long after tissue healing and recovery has taken place (2). It is therefore maladaptive and serves no obvious biological purpose (5, 34, 35).

Many of the investigations into the etiology of neuropathic pain involve controlled, traumatic perturbations leading to defined and reproducible injuries to the spinal cord or peripheral nerves. Surgical, chemical or genetically-induced lesions to rodent peripheral neurons are followed by *in vivo* or *ex vivo* investigations of the properties of primary afferent, spinal or supra-spinal neurons. These are correlated with behavioral studies that seek to assess pain intensity by indices such as thermal or mechanical allodynia and hyperalgesia (36–40). Improvements in behavioral approaches within the last 15 years have focused on assessing pain in terms of its accepted definition as “An unpleasant sensory and emotional experience associated with, or resembling that associated with, actual or potential tissue damage,” (41). Thus, contemporary operant models seek to provide quantification of pain *per se* as opposed to nociception (39). For example, rodents may be required to make a conscious choice between being in a pain-inducing environment and an otherwise undesirable environment such as a brightly illuminated space (4, 42–44). The time spent in the undesirable environment gives an index of the pain the animal is experiencing. A complementary approach to pain quantification involves assessment of behaviors such as social interaction, nest-building, ultrasonic vocalization, burrowing behavior and “facial grimace score” (45–47).

**Figure 1:**
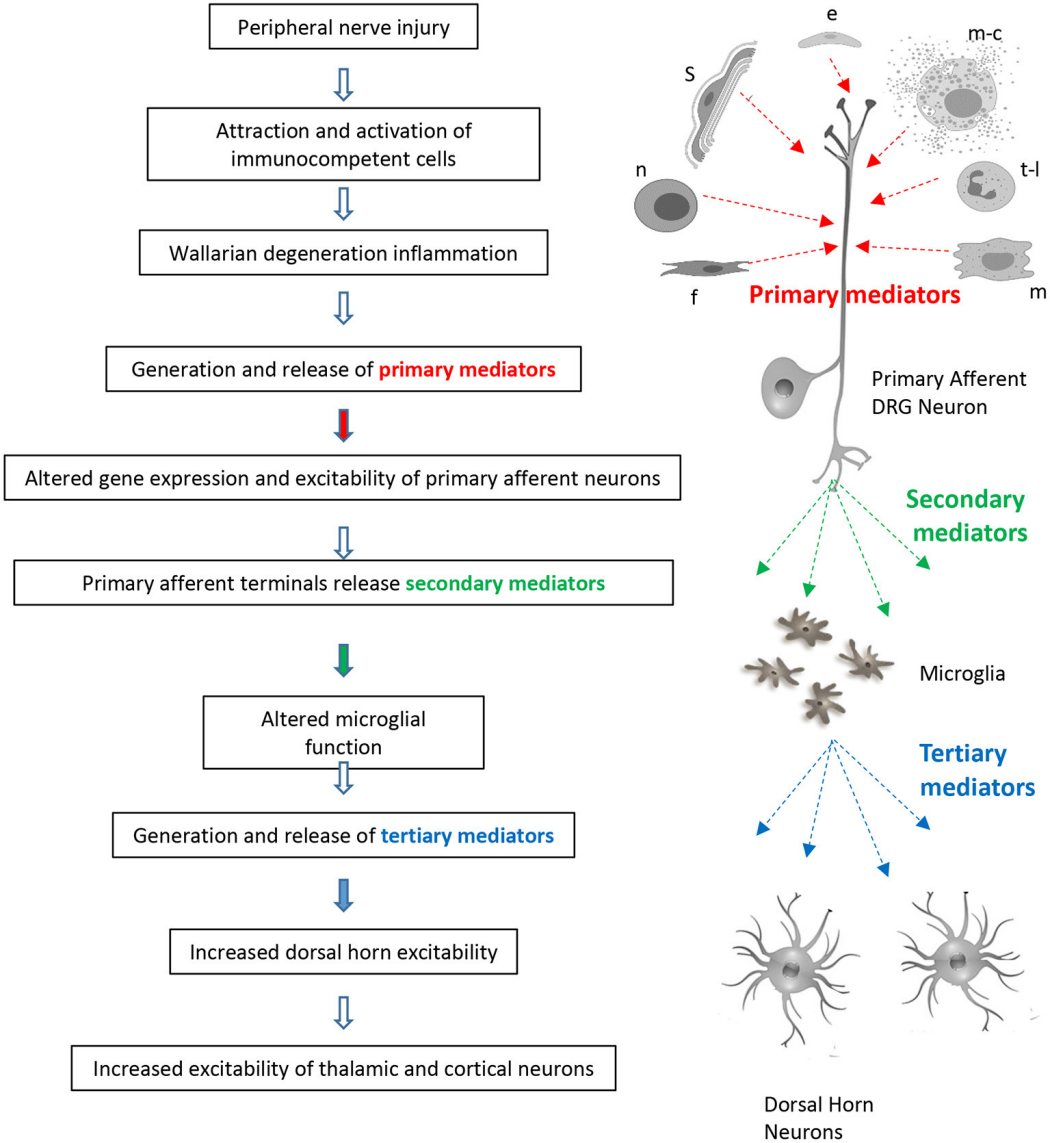
Sites of action of primary, secondary, and tertiary mediators in signaling of neuropathic pain. Sources of primary mediators include Schwann cells (s), epithelial cells (e), mast cells (m-c), t-lymphocytes (t-l), macrophages (m), fibroblasts (f), and neutrophils (n).

Regardless of the methodology used to assess the behavioral consequences of peripheral nerve injury, it is generally accepted that;

1. Peripheral nerve injury promotes Wallerian degeneration of severed axons, macrophage, neutrophil and T-lymphocyte invasion, Schwann cell, fibroblast, mast cell, and epithelial cell activation and the generation of an “inflammatory soup” containing **primary mediators** such as chemokines, cytokines, Wnt ligands, neuropeptides, and growth factors (see Table 1 and Figure 1).
2. Primary mediators sensitize sensory nerve endings, attract additional macrophages and lymphocytes, alter gene expression, promote post-translational modification of proteins, and alter ion channel function in primary afferent neurons. This leads to increased excitability, spontaneous activity and the generation of secondary mediators (see Table 2 and Figure 1).
3. **Secondary mediators** such as colony stimulating factor 1 (CSF-1), chemokine (C-C motif) ligand 21 (CCL21), and wingless-type mammary tumor virus integration site family, member 5A (Wnt5a) are released from primary afferent terminals in the spinal dorsal horn. They affect the properties of spinal microglial cells causing them to release tertiary mediators. In this way, spinal microglia can detect and respond to peripheral nerve injury.
4. Microglial-derived **tertiary mediators** such as BDNF, TNF-α, and IL-1β (Brain derived neurotrophic factor, tumor necrosis factor alpha, and interleukin-1β) increase excitatory transmission and attenuate inhibitory synaptic transmission in the superficial dorsal horn (see Table 3 and Figure 1).
5. This and other aspects of synaptic plasticity facilitate the transfer of nociceptive information and promote misprocessing of sensory information leading to central sensitization at both the spinal and supra-spinal level.
6. Although it was once believed that altered microglial function was transient and confined to the onset phase of neuropathic pain, newer data implicates sustained alteration of microglial function in its long term maintenance. This is associated with long-term changes in astrocyte function.
7. Cell type involvement is sex dependent. Whereas, microglia play a predominant role in central sensitization in males, this is effected by macrophages and T-lymphocytes in females.
8. In addition to release of mediators, recent evidence suggests that cell to cell communication may be affected by the transfer of materials such as microRNA's in secreted extracellular vesicles or exosomes.

**Table 1:** Primary mediators from site of nerve injury.

**Primary mediator**	**Generated and/or released by injured peripheral tissue**	**Mimicking neuropathic pain *in vivo***	**Alleviation of neuropathic pain in knockouts or by antagonists etc. *in vivo***	**Demonstrated effect on dorsal root ganglion neurons**
IL-1β	(48–54)	(55, 56)	(48, 57–60)	(61–65)
IL-15	(66)	(67)^*^		
IL-17	(68, 69)	(21, 70)	(21, 71, 72)	(21)
IL-18			(73)	
LIF	(74, 75)	(75)		(76–78)^†^ (79)^††^
TNF-α or β	(48, 50, 51, 54, 80–82)	(48, 54, 55, 80, 83)	(48, 52, 83–87)	(83, 88–92)
Prostaglandins and other eicosanoids	(54, 93, 94)^**^ (95)^***^	(96) (95)^***^	(94)	(97, 98) (95)^***^
NGF	(99)	(100, 101)	(99, 100, 102, 103)	(101, 104)
Substance P	(105, 106)	(107)	(108, 109)	(110–112)
MCP-1/CCL2	(49, 52, 113–116)	(116)	(52, 116, 117)	(116, 118, 119)
CXCL-1	(120, 121)			(122–124)
CXCL-4	(125)		(123, 125, 126)	(125)
Histamine	(127)	(128)	(128–130)	(128, 130)
Wnt3a	(131–134)	(133)	(131, 133)	(131, 133)
Wnt5a	(135)			

*Released from Site of injury and affect primary afferent neurons*.

* *Implied from observations on osteoarthritis patients*.

** *Measured increased cyclo-oxygenase 2 (COX 2) levels*.

*** *This work addresses the actions of the novel eicosanoid 5,6 epoxyeicosatrienoic acid (5,6 EET)*.

† *These 3 papers show LIF promotes sprouting of perivascular sympathetic axons in DRG*.

†† *This paper demonstrated a direct action of LIF on DRG neurons*.

**Table 2:** Secondary mediators released from primary afferents.

**Secondary mediator**	**Generated and/or released by DRG neurons**	**Mimicking neuropathic pain *in vivo***	**Alleviation of neuropathic pain in knockouts or by antagonists etc. *in vivo***	**Demonstrated effect on microglia**
CSF-1	(136–142)	(138)	(138, 139)	(138–140) (143)^*^
CCL21	(144–146)	(144, 147)	(144, 147–149)	(144, 148)

*Released from primary afferents to affect spinal microglia^†^*.

* *This paper provides indirect evidence, CSF-1 releases BDNF from microglia as monitored by increased dorsal horn excitability, some of the effect of CSF-1 on excitability is abrogated by BDNF binding protein*.

*^†^Wnt3a may be released from primary afferent terminals after nerve injury but is thought to signal directly to dorsal horn neurons without the intervention of microglia (134)*.

**Table 3:** Tertiary mediators produced by microglia to affect spinal dorsal horn neurons.

**Tertiary mediator**	**Generated and/or released by microglia**	**Mimicking neuropathic pain *in vivo***	**Alleviation of neuropathic pain in knockouts or by antagonists etc. *in vivo***	**Demonstrated effect on spinal dorsal horn neurons**
BDNF	(150, 151)	(152–154)	(150, 151, 153, 155)	(4, 143, 150, 154, 156–165)
IL-1β	(166–168)	(166, 169) (55)^*^	(2, 54, 57–60, 166, 170–174)	(175–179)
TNF-α	(54, 180)	(40, 181)	(84, 203, 277, 386).	(175, 182–184)

*Microglia to spinal dorsal horn neurons*.

* *These experiments involved injection of IL-1β into peripheral nerve, thus its ability to produce allodynia most likely reflected its peripheral role as a primary mediator*.

Each of these steps will be discussed below with special emphasis on the actions of **secondary mediators** on microglial activity and the release and actions of **tertiary mediators** in the spinal dorsal horn (Figure 1). Cytokine/chemokine/growth factor/glial cell interactions are also involved in modulation of sensory information in supraspinal structures following peripheral nerve injury. This includes the mesolimbic system (185) thalamus, sensory cortex, and amygdala (186–188). Interestingly, microglial activation appears on the contralateral side following nerve injury thus reflecting the projections of ascending tracts. Activation is not seen in areas which are not involved in pain processing such as the motor cortex (186). This implies that microglial activation in higher centers is not simply the result of diffusion of messengers *via* the cerebrospinal fluid (CSF). The present review will however focus on microglia activity within the spinal dorsal horn.

## Nerve Injury, Wallerian Degeneration, Inflammation and Generation of Primary Mediators

Wallerian degeneration of injured peripheral nerves is associated with neutrophil, macrophage and T-lymphocyte infiltration, mast cell, endothelial cell, keratinocyte and fibroblast activation and alteration of Schwann cell properties (2, 54, 68, 80, 98, 189–196). All of these cell types produce and release a variety of inflammatory mediators and a few anti-inflammatory agents at the site of injury (2, 190, 197) and Table 1. These **primary mediators** include pro-inflammatory agents such as interleukin 1β (IL-1β) (48–50, 55, 57–59, 141), leukemia inhibitory factor (LIF) (74–76, 79, 198), interleukin 15 (IL-15) (66), interleukin 17 (IL-17) (21, 68, 70), interleukin 18 (IL-18) (199) tumor necrosis factor (TNF-α) (48, 51, 80, 83, 85–88, 200–203), monocyte chemoattractant protein 1 (MCP-1/CCL2) (49, 113–115, 118), chemokine (C-X-C motif) ligand 1 (CXCL1) (120–124) and CXCL4 (125), histamine (127–130), and the secreted glycoproteins Wnt3a (wingless-type mammary tumor virus integration site family, member 3A) and Wnt5a (133, 135). For a more complete list see Moalem and Tracey (54).

As discussed below, most of these mediators excite peripheral nerve endings as well as the cell bodies of primary afferent fibers in the dorsal root ganglion (DRG) (53). Release of pro-inflammatory **primary mediators** both at the site of injury and within the DRG provokes changes in the cell bodies, axons and peripheral endings of both injured and uninjured primary afferent axons (141, 204–206).

Satellite glial cells that surround the cell bodies of dorsal root ganglia (DRG) neurons represent an additional source of primary inflammatory mediators (2, 78, 142, 207–209). IL-1β may also be derived from macrophages that invade DRG after injury (141) as well as from sensory neuron resident macrophages (210). Peripheral nerve injury causes extensive satellite glial cell activation (as defined by glial fibrillary acidic protein [GFAP] immunoreactivity). This is prevented by local perfusion of TTX or bupivacaine. Na^+^ channel block also reduces levels of NGF at a time when activated glia (Schwann cells) are an important source of NGF. This implicates injury-induced increased spontaneous activity in primary afferents in the activation of satellite glial cells (211). This aligns with the general concept of “neurogenic neuroinflammation” whereby intense neuronal activity can orchestrate immune cell activation (212).

In addition to the interactions of inflammatory mediators with neurons, many of them promote plasma extravasation and exhibit chemoattractant properties, both of which enable the recruitment of immunocompetent leucocytes and lymphocytes to the site of injury (54, 66, 68, 194). As already mentioned, these myeloid and lymphoid cells themselves release a host of cytokines and chemokines thereby instigating a positive feedback process in the initiation of neuroinflammation.

Although inflammation is a primary response to tissue injury, it should be noted that some of the primary mediators associated with neuropathic pain also serve to initiate neuronal recovery and repair (213). Thus, production of NGF at the site of nerve injury (99, 100, 102, 103, 214) may be viewed as both an initiator of inflammation and an activator of neuronal regeneration and repair. Moreover, functional recovery after peripheral nerve injury may depend on the pro-inflammatory cytokines IL-1β and TNF (48).

The situation with GDNF family ligands such as artemin is complex, whilst some reports describe its pro-inflammatory action and possible involvement in neuropathic pain, others suggest that artemin may be anti-inflammatory and activation of its receptors provide pain relief (215–219).

Interleukin 4 (IL-4) produced by peripheral nerve injury has exclusive anti-inflammatory and anti-nociceptive actions (220). These findings relate to the generalization that both inflammatory and anti-inflammatory mediators are released by nerve injury and it is disturbance of the balance between these two processes that can lead to pain (197).

### Downstream Effectors of Mediator Actions

Although receptors for individual cytokines are selective for their respective ligands, the downstream transduction pathways often converge, resulting in translocation of transcription factors to the nucleus and transcription of additional downstream mediators. Common signaling pathways activated following cytokine receptor activation include (1) nuclear factor-κB (NF-κB), (2) the mitogen-activated protein kinases (MAPKs), (3) the janus kinase (JAK) and signal transducer and activator of transcription (STAT), and (4) the Smad family signaling pathways (50, 187).

By contrast, chemokines, histamine and neuropeptides such as substance P signal *via* heptahelical G-protein coupled receptors (221, 222).

At least some of the actions of inflammatory cytokines involve activation of cyclo-oxygenase 2 (105, 223, 224) and products such as prostaglandins (93, 94, 167, 180, 225, 226) and prostacyclin (227).

Wnt ligands (Wnt; wingless-type mammary tumor virus integration site family) are a family of 19 secreted glycoproteins that are important and versatile mediators of cell–cell communication, cell morphology and development. Ligands signal by the canonical Wnt pathway, the non-canonical planar cell polarity pathway, and the non-canonical Wnt/calcium pathway (133, 228). Wnt3a acts through the canonical pathway which involves β catenin. Wnt5a acts through the non-canonical β catenin independent planar cell polarity pathway and the Ryk (134).

The downstream mediators of BDNF activation of TrkB and NGF activation of TrkA are well-characterized and include the phosphatidyl inositol-3 (PI3)-kinase (also known as Akt or protein kinase B), phospholipase C-γ1 and the ras-MAPK pathway, also known as the extracellular receptor kinase (ERK) pathway (229). Since ras-MAPK is a mediator of both neurotrophin and cytokine receptor activation, there is considerable interest in its potential as a drug target (230–232).

## Effects of Primary Mediators on Primary Afferent Neurons

Gene array analysis of perturbations in primary afferent neurons following nerve injury have identified marked changes in genes coding for neuropeptides, cytokines, chemokines, receptors, ion channels, signal transduction molecules and synaptic vesicle proteins (146, 233) as well as changes in expression of long non-coding RNA's (234) and microRNA's (235–238). The latter post-transcriptionally regulate the protein expression of hundreds of genes in a sequence-specific manner (239). For example the microRNA (miRNA-let-7b) can be released from DRG neurons by neuronal activation. It acts in a paracrine function to induce rapid inward currents and action potentials in other DRG neurons by inducing toll like receptor 7 (TLR7)/TRPA1-dependent single-channel activities. Intraplantar injection of miRNA-let-7b elicits rapid spontaneous pain *via* TLR7 and TRPA1 (240). These observations again align with the concept of neurogenic neuroinflammation (212).

In addition, miR-21-5p which is released in the exosomal fraction of cultured DRG neurons, may be involved in neuron-macrophage communication after nerve injury (238, 241). The concept of cell-to-cell transport of material *via* exosomes or extracellular vesicles represents an exciting new direction for pain research (238, 241–245). A recent review focussed on release of extracellular vesicles from microglia (246).

### Changes in DRG Excitability and Ion Channel Function

Recordings from rodent DRG neurons both *ex vivo* and *in vitro* confirmed that peripheral nerve injury increases their excitability and may provoke spontaneous discharge of action potentials (247–253). This peripheral sensitization and ongoing, aberrant spontaneous activity is a well-established harbinger of central sensitization and chronic pain (5, 7, 9, 251, 252, 254–260). Spontaneous activity is also known to promote activation of spinal microglia and astrocytes (211, 212). Suppression of this activity *in vivo* by either pharmacological (257, 261) or optogenetic methodologies (262) leads to abatement of injury-induced allodynia and attenuation of hyperalgesia.

Increased DRG excitability is driven by increased expression and/or function of voltage-gated Na^+^, Ca^2+^ and hyperpolarization activated cyclic nucleotide gated channels (HCN channels) (263–265) as well as decreased expression and/or function of K^+^ channels (260) and altered expression, modulation and function of acid sensing ion channels (ASIC channels) and transient receptor potential (TRP) channels including TRPV1, TRPA1, and TRPM8 (215, 266–268).

Acute and/or long term exposure of DRG neurons to pro-inflammatory primary mediators such as IL-1β (interleukin 1β), IL-17 (interleukin 17), TNF (tumor necrosis factor), MCP-1/CCL-2 (monocyte chemoattractant protein-1/chemokine ligand 2), stromal cell-derived factor 1 (CXCL12), Wnt3a or prostaglandin E2 increases their excitability (21, 61, 64, 65, 88, 91, 116, 118, 119, 123, 125, 131, 133, 269, 270).

In general, the effects of primary mediators on cation channel function parallel the changes provoked by peripheral nerve injury (62, 63, 92, 125, 184, 271, 271–274) and it is now well-established that these excitatory actions play an indispensable role in the development and/or persistence of neuropathic pain. For example, administration of antibodies to interleukin I-receptor (IL-lR) or its genetic deletion or overexpression of interleukin receptor antagonist (IL-RA) reduce pain behavior in mice with experimental neuropathy thereby implicating IL-1β in the onset of neuropathic pain (2, 54, 57, 58, 202). Although IL-1β is involved at several points in the sensory system following nerve injury (176, 179, 187, 275, 276), its peripheral actions are underlined by the observation that local microinjection of recombinant IL-1β at the site of sciatic nerve injury in IL-1β-knock-out mice lowers mechanical pain thresholds to levels observed in injured wild-type animals (48).

The role of IL-17 has been studied in the paclitaxel model of chemotherapy induced pain. In addition to increasing DRG excitability, both IL-17 and paclitaxel facilitate sEPSC activity and attenuate sIPSC activity in the lamina II outer of the mouse dorsal horn. Selective knockdown of IL-17R in certain dorsal horn cells reduces paxlitaxel-induced hypersensitivity. Taken together these findings provide strong support for a role for IL-17 in this type of chronic pain (21).

Actions and involvement of TNF-α as a primary mediator very much parallel those of IL-1β Levels of TNF-α are elevated in sciatic nerve after injury (82, 85) and Nadeau et al. (48) showed that microinjection of TNF-α into TNF-knock-out mice lowered mechanical pain threshold in a similar fashion to IL-1β TNF-α also upregulates Na_v_1.7 in DRG (89) and inhibition of TNF-α signaling results in attenuation or accelerated recovery from injury induced neuropathic pain (52, 84, 277). TNF-α receptors are also upregulated (84). Unlike IL-1β, TNF-α does not appear to participate in macrophage to DRG neuron signaling (141) but like IL-1β actions of TNF-α are not confined to the peripheral nervous system (180, 187, 277).

Although IL-6 is markedly upregulated in the peripheral and central nervous systems following nerve injury (50–52, 278, 279) and is released by macrophages at the site of nerve injury (51, 279), it fails to affect DRG excitability (53) yet has been reported to attenuate peripheral nociceptive transmission (280). This contradicts the finding that sciatic chronic constriction injury (CCI) failed to induce hypersensitivity to cutaneous heat and pressure in mice with a null mutation of the IL-6 gene (281). Its potential role as a primary mediator thus remains to be resolved. One possibility is that IL-6 serves as an “off signal” to ensure the transient nature of injury-induced neuroinflammation. It may fulfill this function in the spinal cord where it promotes a desensitized phenotype of microglia (282). Some lines of evidence implicate IL-15, IL-17, and IL-18 as primary mediators in the generation of neuropathic pain (Table 1).

Wnt3a also increases sensory neuron excitability *via* upregulation of P2X3 and TRPA1 receptor channels and stimulates production of inflammatory cytokines such as TNF-α and IL-18. Intraplantar injection promotes mechanical hypersensitivity and thermal hyperalgesia. These effects are prevented by inhibition of disheveled; one of the downstream effectors of Wnt3a action (133). Nerve injury also provokes the release of Wnt5a from Schwann cells and since its cognate receptors are upregulated in DRG neurons (135), it, like Wnt3a, may serve as a primary mediator in the onset of neuropathic pain.

Appearance of ectopic excitatory α-adrenoceptors and sprouting of perivascular sympathetic axons both within DRG and on nerve terminals at the site of injury is yet another means by which primary afferent excitability is increased (283–287), leading to signs of neuropathic pain in animal models (288). Sympathetic-sensory interaction is a characteristic feature of complex regional pain syndromes in humans (289). This may reflect a neurotrophic action of LIF or NGF on noradrenergic perivascular axons (76–78) and/or may be a consequence of spontaneous afferent activity (290).

### Changes in Expression of Cytokines, Wnt Ligands, and Neuropeptides in Primary Afferent Neurons; Primary Mediators Promote Production of Secondary Mediators

#### Neuropeptides

Nerve injury alters expression of neuropeptides and their cognate receptors in DRG cell bodies (291–293). Studies have focussed on galanin, NPY, calcitonin gene related peptide (CGRP) and substance P. Since there is evidence for a role of a diffusible substance in soma—soma interactions (294), neuropeptides may play a role in controlling DRG excitability (295). For example, substance P is released in a Ca^2+^ dependent manner from DRG cell bodies (296) and its expression is increased after nerve injury (106, 297). Because large DRG neurons start to express excitatory substance P receptors after nerve injury, it may well play a role in pain etiology (298). This is because alterations in the properties of large DRG neurons and their associated low threshold Aβ fiber axons play major role in neuropathic pain (249, 299–303).

CGRP is also released in DRG where it may fulfill an excitatory autocrine and/or paracrine function in a similar fashion to substance P (122, 295, 304, 305).

#### Chemokines, Cytokines, and Wnt Ligands

Nerve injury upregulates mRNA and/or protein for a variety of secreted proteins, including chemokines, Wnt ligands, and cytokines and/or their receptors in primary afferent neurons. This includes IL-6 and its receptor (209, 278), MCP-1/CCL2 and CC chemokine receptor 2 (CCR2) (270, 306–308), TNF-α (309), IL-1β and IL-10 (306, 310), CCL-21 (146), and Wnt5a (134). As will be discussed below, several of these substances are released from primary afferent nerve terminals and serve as secondary mediators in the dorsal horn; conveying altered peripheral activity to microglia and/or to dorsal horn neurons. The weight of the evidence supports a secondary mediator role for CSF-1 and for the chemokine CCL-21 (Table 2).

## Secondary Mediators from Primary Afferent Terminals Alter Function of Spinal Microglia

### Signaling Between Injured Peripheral Nerve and Spinal Microglia

Following nerve injury, several substances generated in and released from primary afferents serve as **secondary mediators** that influence the properties of spinal microglia (238). In this way microglia can detect and mount a response to peripheral nerve injury.

#### Secondary Mediator Role of CSF-1

Injury-induced release of inflammatory mediators such as interleukin 1β from satellite glial cells and invading macrophages in DRG induces *Csf1* in the cell bodies of primary afferent neurons (136, 137, 141, 142). mRNA for colony stimulating factor (CSF-1) is also upregulated by nerve injury as is mRNA for the CSF-1 receptor in spinal microglia (138). Intrathecal injection of recombinant CSF-1 induces microglial proliferation and renewal as well as mechanical allodynia in naïve animals (138–140). When *Csf1* gene expression is selectively depleted from sensory neurons, nerve injury-induced CSF-1 expression and the development of mechanical hypersensitivity are prevented as is the injury-induced microglial activation and proliferation (141).

Release of CSF-1 from primary afferent terminals transforms the phenotype of resting microglia such that they expresses the ionotropic ATP receptor, P2X4R (138, 139, 143). The membrane adaptor protein DAP12 is required for nerve injury-induced upregulation of P2X4R but not for microglial proliferation. Taken together, with the observation that long term exposure of dorsal horn neurons to CSF-1 increases their excitability (143), these data support its role as a secondary mediator signaling between injured primary afferents and microglia which then release **tertiary mediators** such as BDNF and IL-1β (150, 157, 311).

ATP derived from dorsal horn neurons activates P2X4 receptors on microglia, promoting Ca^2+^ influx and BDNF release (151, 312–318). As will be discussed below, this mechanism is crucial to glial signaling and the development of central sensitization in males (313, 319) but not in females (27, 320).

#### MCP-1/CCL2 Plays a Neuromodulatory Role Within Injured DRG but Is Unlikely to Function as a Secondary Mediator Between Nerves and Microglia

Mice lacking the CCR2 receptor for the chemokine MCP-1/CCL-2 fail to develop signs of neuropathic pain following nerve injury (118, 321), a MCP-1/CCL2 antagonist blocks paclitaxel-induced neuropathic pain (52) and over expression of CCR2 enhances nociceptive responses (322). MCP-1/CCL2 is not found in undamaged peripheral nerves but is strongly upregulated following injury (221, 323). This may be a consequence of the action of TNF-α and spontaneous neural activity (118, 324). MCP-1/CCL2 is expressed in vesicles in DRG soma (117, 270) and is released from DRG cell bodies in a Ca^2+^ dependent manner (270). This evoked release is increased under neuropathic conditions (115, 325). Injury has also been reported to increase immunoreactivity for CCR2 in dorsal horn microglia (326) and spinal administration of CCL2 promotes microglial activation (325, 327). Although these findings might be expected if MCP-1/CCL2 serves as a secondary mediator between primary afferents and spinal microglia, recent work casts doubt on this conclusion. For example, Jung et al. (117) did not detect MCP-1/CCL2 in primary afferent terminals and other studies of microglia *in vivo* failed to confirm the presence of CCR2 either before or after nerve injury (117, 146, 328). Now that more specific biomarkers for cell types are available, one possible explanation for this discrepancy is that CCR2 may be expressed on infiltrating monocytes or on astrocytes rather than on microglia (221, 329, 330).

Rather than functioning as a secondary mediator between primary afferents and spinal microglia, MCP-1/CCL2 may fulfill an autocrine or paracrine function within DRG (118, 270). This possibility is supported by the aforementioned observation that MCP-1/CCL2 is released from DRG cell bodies in a Ca^2+^ dependent manner (270). It has also been shown to excite injured DRG neurons by transactivation of TRPA1 and TRPV1 channels (115, 118). MCP-1/CCL2 may thus stimulate first order neurons in the pain cascade and/or carry out its classical chemokine function to attract CCR2-expressing peripheral monocytes/macrophages to the spinal cord (117, 146). MCP-1/CCL2 may also promote the release of the excitatory neuropeptide CGRP within DRG (122).

#### Secondary Mediator Role for CCL-21

Intrathecal administration of chemokine (C-C motif) ligand 21 (CCL21) rapidly induces pain-like behavior in naive mice whereas CCL21 neutralizing antibodies or blockade of its cognate CXCR3 receptors with (+/–)-NBI-74330 diminishes pain-related behavior in nerve injured animals (147). The failure of CCL21 deficient mice to display tactile allodynia following nerve injury (148) has been ascribed to the failure of microglia to upregulate the P2X4 receptor for ATP (144, 146). CCL21 is upregulated in DRG following nerve injury, vesicles containing CCL21 are preferentially transported into axons (145), CCL21 affects microglial function (148) and it can be released from terminals of injured or “endangered” neurons (149, 331). Taken together, these findings suggest that CCL21 is more likely than MCP-1/CCL2 to function as a secondary mediator between primary afferents and microglia following injury (146, 221). CCL21 has also been reported to signal to astrocytes (332).

#### What Is the Role of Stromal Cell-Derived Factor-l Alpha (CXCL-12/SDF-lα)?

Stromal cell-derived factor-l alpha (SDF-lα) also known as C-X-C motif chemokine 12 (CXCL12), and its cognate receptor CXCR4, are constitutively present in DRG neurons and satellite glia, spinal astrocytes and microglia (333, 334). Peripheral nerve injury upregulates both CXCL12 and CXCR4 in DRG and/or spinal cord (123, 221, 333, 335, 336) as a possible consequence of the action of TNF-α (336). The functional significance of these changes is demonstrated by the observation that CXCL12-induced Ca^2+^ response in DRG neurons is enhanced in nerve injured animals (123). Intrathecal administration of CXCL12 induces hypersensitivity in naive rats in a CXCR4 dependent manner (333, 333). In addition intrathecal injection of CXCL12 neutralizing antibody or the CXCL12 antagonist, AMD3100 transiently reverses allodynia after peripheral nerve injury (123, 336).

CXCL12 has been implicated in pain signaling following spinal cord injury (337) and may be involved in hyperalgesic priming (338). In view of this and the findings presented above, it is clear that the CXCL12–CXCR4 system has an important role in modulation of neuropathic pain. It may be particularly involved in astrocyte signaling and long term pain maintenance (333). Despite this, we could find no reports that CXCL12 is released from injured primary afferents to affect microglia. It thus remains to be determined whether CXCL12 functions as a *bona fide* secondary mediator.

#### What Is the Role of Fractalkine (CX3CL1)?

Fractalkine (CX3CL1) is produced constitutively by spinal cord neurons (339, 340) and its receptors (CX3CR1) are primarily expressed by dorsal horn microglia (340, 341). These are upregulated after nerve injury *via* an IL-6 dependent mechanism (342). Intrathecal injection of fractalkine produces mechanical allodynia and thermal hyperalgesia whereas injection of a neutralizing antibody raised against CX3CR1 delays the onset of mechanical allodynia and/or thermal hyperalgesia in two different neuropathic pain models (341). This is consistent with the observation that mice lacking CX3CR1 do not display allodynia following peripheral nerve injury (343).

Fractalkine exists in both a membrane tethered form and as a soluble protein (344). Nerve injury increases the level of soluble fractalkine in cerebrospinal fluid (345) and this release by cathepsin S appears obligatory for the expression of neuropathic pain (221, 346). Soluble fractalkine promotes microglia activation and the generation of tertiary mediators including IL-1β and TNF (167, 341).

Cathepsin S is itself released from microglia by an ATP-P2X7 dependent mechanism (347). Since fractalkine immunoreactivity does not localize with CGRP, IB4 or NF200 in the dorsal horn, it has been suggested that under neuropathic conditions, stimulation of primary afferent fibers induces release of cathepsin S from microglia, which liberates soluble fractalkine from dorsal horn neurons, thereby contributing to the amplification and maintenance of chronic pain (345). Since production of soluble fractalkine requires prior release of cathepsin S from already activated microglia, it cannot be regarded as a straightforward secondary mediator, signaling between neurons and microglia in the same way as CCL21 or CSF-1.

Because antibodies raised against CX3CR1 reduce nociceptive responses when administered 5–7 days after CCI, the prolonged release of fractalkine may contribute to the maintenance as opposed to the onset of neuropathic pain. This may relate to the observation that nerve injury provokes *de novo* expression of CX3CL1 in dorsal horn astrocytes (340).

Fractalkine signaling has also been implicated in synaptic degeneration seen in HIV patients who experience painful neuropathy (8). This can be modeled in mice by intrathecal injection of the viral coat protein gp120. This upregulates fractalkine and knockout of its cognate receptor CX3CR1 protects synapses from gp120-induced toxicity. Inhibition of the Wnt/β-catenin signaling blocks both gp120-induced fractalkine upregulation and synaptic degeneration. Injection of gp120 stimulates Wnt/beta-catenin-regulated fractalkine expression *via* NMDA receptors and the NMDA antagonist APV, Wnt/beta-catenin signaling suppressor DKK1, or knockout of CX3CR1 alleviate gp120-induced mechanical allodynia. Taken together the results suggest that HIV-1 gp120 provokes synaptic degeneration in dorsal horn by activating microglia *via* Wnt3a/beta-catenin-regulated fractalkine expression.

#### What Is the Role of Interferon Gamma?

Several lines of evidence implicate interferon gamma (IFN-γ) in the etiology of neuropathic pain. Spinal microglia in naive animals express the appropriate receptor (IFN-γR) and stimulation with IFN-γ induces both tactile allodynia and altered microglia function. Genetic ablation of IFN-γR impairs nerve injury-evoked activation of ipsilateral microglia and tactile allodynia (348). The purinergic P2X4 receptor is upregulated in IFN-γ stimulated—microglia and, as will be discussed below, the appearance of such receptors plays a crucial role in the onset of neuropathic pain in males (151, 312, 314, 316, 317). IFN-γ has also been shown to increase dorsal horn excitability (349, 350) and to facilitate synaptic transmission between C-fibers and Lamina 1 neurons *via* a microglial dependent mechanism (351). Although the level of IFN-γ is increased in spinal cord following peripheral nerve injury (352), this may originate from invading T-lymphocytes. This implies that IFN-γ does not have a major role as a secondary mediator to effect communication between injured primary afferents and microglia.

### Microglial-Independent Signaling Between Primary Afferents and Dorsal Horn Neurons

Apart from the role of glutamate and its involvement in long term potentiation (353), there are several situations where secondary messengers generated in, and released from primary afferents exert direct long term effects on dorsal horn neurons. For example, the primary mediator role of the secreted glycoprotein Wnt3a has already been alluded to Simonetti et al. (133). Recent evidence suggests that Wnt3a promotes the release of another ligand, Wnt5a from primary afferents which in turn promotes dendritic retraction of dorsal horn neurons (134). This occurs without the intervention of microglial signaling.

The secondary mediator CSF-1 decreases excitatory drive to inhibitory neurons in dorsal horn *via* a BDNF independent process (143). Since the presence of CSF-1 receptors on neurons has been questioned (354), it remains to be determined whether this reflects a direct effect of CSF-1 on neurons or whether other microglial derived tertiary mediators are recruited.

## Release of Tertiary Mediators From Microglial Cells

### Release of BDNF in the Spinal Dorsal Horn

Initial studies on the release and actions of BDNF were predominantly done on male rodents in an attempt to avoid possible complications imposed by the female oestrous cycle. More recent data strongly suggest major differences in the mechanism of central sensation in females compared to males; microglial derived BDNF is probably not involved in females (24, 26, 27, 313, 320, 355). In males however, numerous lines of behavioral and cellular data strongly implicate the release of BDNF from spinal microglia in the etiology of neuropathic pain (4, 143, 150, 152, 154, 156, 157, 161, 164, 315, 356–359).

As already mentioned, the secondary mediator CSF-1 is released from injured primary afferents and interacts with its receptors on microglial cells (137). This leads to the up regulation of several genes that are implicated in the development of neuropathic pain. This includes *Itgam* (encoding CD11b), *Cx3cr1* (encoding the fractalkine/CX3CL1 receptor, CX3CR1), *Bndf* (encoding BDNF), and *Ctss* (encoding cathepsin S) (139). BDNF which acts by increasing dorsal horn excitability, is a major tertiary mediator in the development of central sensitization (4, 143, 150, 151, 156, 157, 163, 314, 315).

Long-term exposure of dorsal horn neurons to CSF-1 also increases their excitability and this effect is abrogated by the BDNF binding protein TrkB-fc (143). These findings underline the importance of a sensory neuron—CSF-1—microglia—BDNF signaling process in the onset of neuropathic pain (4, 9, 139, 238, 360).

#### Role of ATP and P2X4 in BDNF Release

Although stimulation of primary afferents releases ATP and generates P2X mediated EPSC's in a subpopulation of lamina II neurons (361), primary afferent neurons do not appear to be the main source of ATP following peripheral nerve injury. It may rather derive from neurons in the superficial dorsal horn itself (362). BDNF release from microglia is brought about by ATP activation of upregulated P2X4R (151, 168, 312, 314, 316–318). This release is biphasic. An early phase occurs within 5 min, whereas a late phase peaks 60 min after ATP stimulation. The late phase of release is associated with an increased level of BDNF within the microglia. Both phases of BDNF release are dependent on extracellular Ca^2+^ but the late phase of release and accumulation is dependent on transcription and translation. This suggests that activation of P2X4R initially releases a pre-existing pool of BDNF and subsequently promotes *de novo* synthesis of BDNF. This vesicular release of BDNF is abolished by inhibiting SNARE (soluble N-ethylmaleimide-sensitive factor attachment protein receptor)-mediated exocytosis and the P2X4R-evoked release and synthesis of BDNF are dependent on activation of p38-mitogen-activated protein kinase (MAPK) (312, 314–317).

Activation of P2X4 on microglia and release of BDNF are involved in the onset of neuropathic pain in males, but as already mentioned, not in females. This is congruent with the observation that spinal microglia from female rodents do not express P2XR (26).

#### Role of ATP and Metabotropic P2Y Receptors in BDNF Release

There is also evidence for a role for metabotropic P2Y receptors in microglial activation and the onset of neuropathic pain (363–365). This involves P2Y6, 11, 12, 13, and 14 (366–369). Whilst P2Y6 signals through G_q/11_ and P2Y12, 13, and 14 signal through G_s_, P2Y11 signals through both G_q_ and G_s_ (222).

P2Y12 mRNA and protein are increased in microglia after peripheral nerve injury and intrathecal injection of a P2Y12 antagonist or antisense knockdown of P2Y12 expression suppresses the development of injury-induced pain behaviors and the phosphorylation of microglial p38 MAPK. By contrast, intrathecal infusion of a P2Y12 agonist into naive rats mimics the nerve injury-induced activation of microglial p38 and increases pain behaviors (366). Since phosphorylation of p38MAPK by P2X4 agonists has been implicated in BDNF release (314) this may also be affected by P2Y12 activation.

Spared nerve injury also induces a p38MAPK-dependent increase in P2Y6, 13, and 14 mRNA in microglia. This is thought to depend on activation of ROCK Rho-associated coiled-coil-containing protein kinase (370). Since intrathecal injection of the specific P2Y6 antagonist MRS2578, the specific P2Y13 antagonist MRS2211 or P2Y14 antisense, attenuate mechanical pain hypersensitivity, these three receptors may function as downstream effectors that mediate some of the actions of ATP in microglia (367, 371).

#### Wnt Signaling and BDNF Release

Wnt signaling can also promote BDNF release (359, 372). This phenomenon has been examined in models of HIV pain which involve exposure of sensory neurons to viral coat proteins such as gp120 (12, 372). Intrathecal injection of gp120 produces mechanical allodynia and increases expression of Wnt3a, β catenin and microglial BDNF in the murine spinal cord. Blockade of Wnt or BDNF signaling alleviates mechanical allodynia as does inhibition of microglial activation with minocycline (12). Zhang et al. (359) have suggested a mechanism whereby Wnt signaling provides an important link between increased neuronal activity and BDNF expression. Increased glutamatergic neuronal activity activates NMDA receptors and increases the level of intraneuronal Ca^2+^ This promotes Wnt protein synthesis and release *via* MAPK/CREB signaling (373, 374). Activation of frizzed receptors on microglia promotes Wnt signaling *via* β catenin leading to increased BDNF expression and release. This is a further illustration of the concept of “neurogenic neuroinflammation” whereby intense neuronal activity promotes immune cell activation (212).

#### BDNF in Inflammatory vs. Neuropathic Pain

Inflammatory pain as induced by formalin or carrageenan exposure is attenuated using the Cre-loxP system to *selectively* delete BDNF from nociceptive sensory neurons. Despite this, these animals display normal signs of neuropathic pain following nerve injury (375). Whilst BDNF thus appears to be involved in both inflammatory and neuropathic pain (376), in the first case it is derived from peripheral nociceptors whereas in the second case it is derived from ATP-activated microglia.

#### Time Course of Microglia Activation and Long-Term Effect of BDNF

Whereas, early studies of microglia activation in response to peripheral nerve injury focussed on short term changes (312), more recent work has shown that microglial activation in rodent dorsal horn persists for more than 3 months after injury (377). Activation even persists beyond the known involvement of pro-inflammatory cytokine signaling. Thus, selective depletion of spinal microglia with the targeted immunotoxin Mac1-saporin or sequestration of BDNF with the selective binding agent TrkBFc almost completely reversed thermal and mechanical alloynia in both the acute (2 week) and chronic (3 month) phase after injury. By contrast, neutralizing cytokine signaling using intrathecal injection of a cocktail of antibodies against IL-β, TNF-α, and IL-6 significantly attenuated tactile and cold allodynia at 2 weeks but not at 3 months after injury. These findings may have therapeutic relevance as they suggest different mediators should be targeted in the management of acute vs. chronic neuropathic pain (377).

#### BDNF, TrkB, and Antidepressants

It has recently been reported that some antidepressants bind to TrkB and augment BDNF signaling (378). Since the many lines of evidence outlined above implicate BDNF in central sensitization, augmentation of TrkB signaling by antidepressants would be expected to exacerbate pain. Despite this, tricyclic antidepressants and serotonin-noradrenaline reuptake inhibitors are first line treatments in neuropathic pain management (379). The relationship between these disparate observations remains to be studied and resolved.

### Release of IL-β in the Spinal Dorsal Horn

IL-1β plays a modulatory or effector role in nociception in the periphery, dorsal root ganglia, spinal cord and higher centers. These effects assume particular importance in the etiology of neuropathic pain. Corroborative evidence for a role of IL-1β neuropathic pain comes from the observation that inhibition of matrix metalloproteases responsible for IL-1β processing leads to attenuation of pain in a rodent model (170).

Whilst the CSF-1, P2X4-microglia-BDNF pathway is well-characterized, less is known about the release of IL-1β. In the spinal cord, it is produced and released from macrophages, astrocytes and microglia (2, 380, 381). Release from microglia is a consequence of activation of P2X7 receptors (166, 168, 311, 319) and may be provoked by the action of fractalkine (167). In agreement with this, it has recently been reported that the Ca_v_1 channel blocker, cilnidipine blocks microglial P2X7 receptors, impairs IL-1β release and reverses nerve injury-induced mechanical hypersensitivity (173). It has also been suggested that P2X4R interact intracellularly with P2X7R to augment P2X7R-mediated IL-1β release (168).

Release of IL-1β is unlikely to reflect a SNARE dependent process as has been suggested for BDNF (314). IL-1β is known to be processed intracellularly from its inactive pro-form by caspase-1 into its mature bioactive form (382). Release from macrophages and dendritic cells and partially from neutrophils, may be brought about *via* the formation of gasdermin D pores in the cell membrane (382–384). One recent report implicates gasdermin D in IL-1β release from microglia in *Toxoplasma gondii* (parasitic protozoan) infections (385) but it remains to be determined whether a similar mode of release is engaged in neuropathic pain. In this situation, IL-1β release may involve its excocytosis *via* panexin channels (166).

### Release of TNF-α in the Spinal Dorsal Horn

The role of TNF-α as a peripheral primary mediator has already been alluded to and several studies have shown that signs of neuropathic pain may be alleviated by perturbation of TNF-α signaling (84, 203, 277, 386). Several lines of evidence also support a role of TNF-α as a tertiary mediator responsible for signaling between microglia and dorsal horn neurons.

Nerve injury increases levels of TNF-α mRNA in spinal microglia and microglia derived cytokine induces COX2 and PGI2 synthase expression in endothelial cells suggesting that a TNF-α mediated glia-endothelial cell interaction is involved in the generation of neuropathic pain (180).

### Release of Wnt 5a in the Spinal Dorsal Horn

Wnt proteins are upregulated in the spinal cord of various pain models (3, 11, 134, 199). In a very consistent manner as seen in the pathogenesis of HIV-associated pain, Wnt ligands (e.g., Wnt5a) are specifically upregulated in the SDH of “pain-positive” HIV patients (11). By regulating the pathogenesis of gp 120—induced pain, Wnt5a sensitizes pain-processing SDH neurons through the JNK/TNF-α signaling pathway.

## Actions of the Tertiary Mediator BDNF in the Dorsal Horn

In male rats, intrathecal administration the BDNF binding protein TrkB-Fc prevents the development of mechanical allodynia after spared nerve injury (387). Several cellular mechanisms have been implicated in the excitatory actions of microglial-derived BDNF that lead to central sensitization.

### BDNF Increases Excitatory Drive to Excitatory Neurons and Decreases That to Inhibitory Neurons

In rat spinal organotypic cultures, 5–6 d exposure to BDNF increases excitatory synaptic drive to excitatory lamina II neurons whilst decreasing excitatory drive to inhibitory neurons (157, 356). In mice, effects of BDNF are dominated by increased excitatory drive to excitatory neurons. Whereas, presynaptic TrkB and p75 neurotrophin receptors are involved, postsynaptic effects are mediated exclusively by TrkB (143). Whilst the passive and active properties of lamina II neurons such as rheobase, resting potential, input resistance and excitability are little affected (143, 157, 356), the altered synaptic activity is capable of increasing spontaneous action potential discharge in excitatory neurons whilst reducing it in inhibitory neurons (356). Three observations show that these actions of BDNF are relevant to injury-CSF-1-microglia evoked central sensitization. Firstly BDNF—induced changes in synaptic transmission and its lack of effect on the intrinsic excitability of lamina II neurons very much parallel those invoked by peripheral nerve injury (157, 388, 389). Secondly, Ca^2+^ responses evoked by neuronal depolarization are enhanced by BDNF and also by conditioned medium from lipopolysaccharide-activated microglia. The effect of this conditioned medium is attenuated by sequestering BDNF with TrkBd5 (157). Thirdly, the putative microglial modulator CSF-1 increases synaptic excitation of excitatory lamina II neurons in mice and this effect is abrogated by sequestering BDNF with TrkBfc (143) whereas, as already mentioned, CSF-1 reduces excitation of putative inhibitory neurons in a BDNF-independent mechanism, suggesting that injured primary afferents can also signal directly to dorsal horn neurons without the involvement of microglia (143).

### BDNF and NMDA Receptor Function

#### Effects of BDNF on Postsynaptic NMDA Receptors

The BDNF effects alluded to above relate primarily to AMPA receptor mediated transmission as neurons were studied at a holding potential of −70 mV (143, 157, 356, 388, 389). There is however a considerable body of evidence to support a role for altered NMDA receptor function in the etiology of pathological pain. This is supported by the occasional success realized with NMDA blockers such as ketamine in the clinic (390, 391). The link between NMDA receptor function and BDNF was established over 20 years ago by the observation that it enhances excitatory responses to NMDA in rat spinal cord *in vitro* (392). BDNF phosphorylates GluN1 *via* ERK and PKC (393). It also acts through TrkB to phosphorylate the GluN2B subunit by the Src-family kinase Fyn and thereby potentiates excitatory NMDA receptor-mediated currents (165). Interestingly, this potentiation appears to require the coincident BDNF mediated Cl^−^ disinhibition. The exact molecular mechanism of this interaction remains to be elucidated as it does not appear to reflect increased NMDA receptor availability as a result of GABA-induced depolarization (165).

#### Effects of BDNF on Presynaptic NMDA Receptors

BDNF also acts *via* TrkB and a Src-family kinase to potentiate the function of presynaptic NMDA receptors on primary afferent terminals (394). It has been reported that presynaptic NMDA receptors only potentiate glutamate release from primary afferents after nerve injury (395). This further underlines the presynaptic BDNF effect in the development of central sensitization.

### BDNF Decreases Inhibition by Perturbation of Chloride Gradients

Peripheral nerve injury reduces expression of the potassium-chloride exporter (KCC2) in spinal lamina 1 neurons (396, 397). The resulting accumulation of intracellular Cl^−^ causes normally outward, inhibitory GABAergic synaptic currents mediated by Cl^−^ influx to become inward excitatory currents mediated by Cl^−^ efflux (396–398). Since the knockdown of spinal KCC2 in non-injured rats reduces pain thresholds and induces neuropathic pain behaviors, these changes contribute to the pathophysiology of central sensitization (150, 396).

In male rats, BDNF mediates this downregulation of KCC2 (164). Thus, administration of ATP activated microglia, but not control microglia, reproduces the shift in anion gradient seen after nerve injury as does application of BDNF. Also, blocking TrkB or using interfering RNA against BDNF reverses both injury induced pain behaviors and the shift in anion gradient (150). Further analysis of this phenomenon reveals that changes in KCC2 expression in deep dorsal horn neurons are confined to nociceptive neurons that project *via* the spinothalamic tract whereas wide dynamic range (WDR) neurons that are activated by a variety of sensory modalities are unaffected (399). It has also been shown that neurons in lamina I are more susceptible to changes in Cl^−^ gradient than those in lamina II (397) and biophysical and modeling analysis shows this loss is especially effective in promoting increased neuronal firing (400). These are important observations as lamina I and deep dorsal horn nociceptive neurons are the most important sites for relay of nociceptive information to the brain (303, 401, 402). Since loss of GABAergic inhibition enables non-noxious Aβ fiber-mediated excitatory transmission to acess the superficial spinal dorsal horn, this process plays a major role in the establishment of allodynia (300, 403, 404).

Reversal of the Cl^−^ gradient may rationalize the observation that BDNF increases GABA release in the dorsal horn (159, 161, 405). Under these conditions GABA produces inward currents (396) which would be enhanced and therefore strongly excitatory.

### BDNF and Induction of Long-Term Potentiation

Long term potentiation (LTP) of synaptic transmission contributes to central sensitization in the dorsal horn (353, 406–408). LTP of C-fiber responses can also be augmented by BDNF (387) and LTP induced by high frequency nerve stimulation is occluded by BDNF treatment (409). This reflects functional upregulation GluN2B subunits of NMDA receptors by activation of the tyrosine phosphatase SHP2 (409) or Fyn kinase-mediated phosphorylation of GluN2B subunit at tyrosine 1472 (387). These authors also showed intrathecal administration of BDNF scavenger TrkB-Fc prior to surgery could prevent the nerve injury-induced increase of both phosphorylated Fyn and phosphorylated GluN2B expression and as mentioned above it also prevented the development of mechanical allodynia after spared nerve injury. The importance of these effects was recently underlined by the observation that spinal LTP induced by high frequency stimulation as well as microglial activation and upregulation of BDNF are inhibited by antibodies to CSF-1. This strongly implicates CSF-1/nerve injury driven microglial derived BDNF in the generation of spinal LTP (408).

### BDNF, Intracellular Ca^2+^ Oscillation, and Spontaneous Bursting Activity

Manipulations that increase neuronal excitability can induce synchronous waves in the level of cytosolic Ca^2+^ that propagate across the whole dorsal horn (410–412). Similarly, K^+^-induced depolarization invokes oscillatory activity as monitored by spontaneous field potentials (413). It has also recently been shown that action potential discharge encodes cytosolic Ca^2+^ levels in lamina 1 neurons and even a single action potential can provoke a measurable Ca^2+^ response (414). This implies that spontaneous bursting activity and oscillations of cytosolic Ca^2+^ level may be closely related. Although long term application of BDNF does not change the resting membrane potential, input resistance of rat dorsal horn neurons in organotypic culture (158) it promotes oscillations in the level of intracellular Ca^2+^ in some neurons whilst depressing it in others (163). There appear to be several mechanisms whereby oscillations may be set up, for example those observed by Alles et al. (163) and Chapman et al. (411) were prevented following blockade of AMPA glutamate receptors whereas those by Asghar et al. (413) were merely attenuated. The oscillations recorded by all three groups were however blocked by TTX, again underlining the tight assocaition between action potential activity and Ca^2+^ signalling which in turn may enable Ca^2+^-dependent gene expression. Whilst the oscillations appeared to be primarily originating from neurons the possible contribution of signal from astrocytes cannot completely be ruled out. Although any direct relationship between these oscillations and neuropathic pain mechanisms remains to be established, sciatic nerve injury has been reported to induce spontaneous bursting activity in a subgroup of dorsal horn neurons *in vivo* (415). MRI studies have also revealed oscillatory activity in the spinal cord of neuropathic pain patients (416). It may be posited therefore that oscillations in Ca^2+^ level and spontaneous bursting activity contribute to the bouts of spontaneous “electric shock like” pain experienced by those afflicted with painful neuropathies (163).

### BDNF in Injury-Induced Synaptic Reorganization in Dorsal Horn Neurons

As already mentioned, peripheral nerve injury produces neuron type specific effects on synaptic transmission in the dorsal horn; excitation of excitatory neurons is increased whereas excitation of inhibitory neurons is decreased (143, 156–159, 356, 388, 389). In addition to altered neurotransmitter release and alterations in postsynaptic sensitivity, connectivity is lost at some synapses (8, 417, 418) but new connections and/or reorganization of dendritic spines occurs at others (408, 419).

Microglia are clearly capable of releasing mediators which promote neuronal loss in an animal model of multiple sclerosis (140) and synaptic degeneration in a model of HIV pain (8). This process of microgliosis is also seen following peripheral nerve injury (420, 421). As discussed below, these processes are likely to reflect the action of microglia-derived BDNF and in the case of HIV pain may reflect phagocytosis of damaged synapses by activated microglia (8).

#### Is BDNF Involved in Injury-Induced Loss of Primary Afferent Terminals Onto Inhibitory Neurons?

Peripheral nerve injury promotes transient loss of glutamatergic excitatory terminals from non-peptidergic IB-4 positive nociceptive fibers in the *substantia gelatinosa* (418, 422). These fibers form the synaptic terminals of the “type 1” synaptic glomeruli (423) which contact GABAergic neurons (402, 424). Morphological changes may therefore contribute to injury-induced reductions in the amplitude and frequency of spontaneous and miniature EPSCs in tonic firing, putative inhibitory neurons (388). This attenuation of excitatory drive to inhibitory neurons would be expected to contribute to an overall increase in dorsal horn excitability (158). Since BDNF also reduces mEPSC amplitude and frequency in putative inhibitory neurons in rat dorsal horn (356) it is possible that BDNF accounts for loss of primary afferent terminals (418, 422). This possibility requires further investigation as BDNF stimulates overall axon growth and regeneration in the spinal cord (425, 426).

This differs from the situation in mice where BDNF does not affect excitatory drive to inhibitory neurons (143). It remains to be determined whether this simply reflects a species difference or whether it is a consequence of the more rigorous criteria to define inhibitory neurons in mice (143, 412) compared to rats (157, 356).

BDNF is not involved in injury-induced loss of GABA terminals. Nerve injury also promotes loss of GABAergic inhibitory terminals in laminae I and II of the dorsal horn (422, 427). Because BDNF enhances GABAergic transmission at various synaptic loci in the dorsal horn (158, 159, 161), the nerve injury-induced loss of inhibitory terminals is unlikely to involve BDNF.

#### BDNF May Increase Primary Afferent Terminals on Excitatory Neurons

In rats, both nerve injury and BDNF increase excitatory synaptic drive to putative excitatory neurons (157, 356, 388, 389, 428) and a similar effect of BDNF is seen in mice. CSF-1 also increases synaptic drive in a BDNF dependent fashion (143). These observations parallel the observation that both BDNF and CSF-1 increase CGRP containing terminals in response to nocigenic high frequency stimulation (408) as these terminals primarily innervate excitatory neurons (402).

### BDNF and Astrocyte Activation

In addition to its actions on neurons as described above, BDNF also activates astrocytes such that they release additional mediators that participate in the establishment of central sensitization (429).

## Actions of the Tertiary Mediator Interleukin 1β in the Dorsal Horn

IL-1β levels are increased in the cerebrospinal fluid (CSF) of patients with complex regional pain syndrome (275) and in spinal cords obtained post-mortem from patients with painful HIV related neuropathy (3). Although there are several reports of the effectiveness of IL-1β antagonists and genetic impairment of cytokine function in animal models of neuropathic pain (57–59, 171) studies of the effectiveness of the modified human interleukin 1 receptor antagonist protein (anakinra) in the clinic have been limited by the pharmacokinetic issues imposed by the blood brain barrier (171).

As mentioned above, release of IL-1β from microglia is primarily affected by activation of P2X7 receptors (166, 173, 311, 319) and/or by the action of fractalkine (167). In a similar fashion to BDNF, IL-1β increases overall dorsal horn excitability, glutamate release from primary afferents and excitatory synaptic transmission between primary afferent C-fibers and lamina 1 neurons (167, 176, 430).

### Effects of IL-1β on Synaptic Transmission in the Spinal Dorsal Horn

Like BDNF, IL-1β does not affect the membrane potential or rheobase of lamina II neurons, suggesting that most of its effect on dorsal horn excitability can be ascribed to changes in synaptic transmission (175, 176). We found that exposing organotypic cultures of rat spinal cord to 100 pM IL-1β for 6–8 d increased the amplitude of spontaneous EPSC's (sEPSC) in putative excitatory “delay” neurons, and decreased the frequency of spontaneous IPSC's (sIPSC). These are somewhat similar to those seen with peripheral nerve injury (388, 389). IL-1β would therefore be expected to increase dorsal horn excitability and to facilitate the transfer of nociceptive information. This was confirmed by the observation that Ca^2+^ responses evoked by exposure of neurons to 20 mM K^+^ were augmented by IL-1β exposure (176). However, other actions of IL-1β included disinhibition of putative inhibitory “tonic” neurons and although the frequency of sIPSC's in putative excitatory “delay” neurons was decreased, their amplitude was increased. The latter observations may be rationalized if GABA assumes an excitatory role in the injury situation due to perturbation of Cl^−^ gradients by BDNF (150).

We used long-term application of IL-1β to parallel the time course of injury-induced changes in spinal cytokine levels (48, 176). Our findings are paralleled by the observations that acute application of IL-1β increases the amplitude of AMPA and NMDA currents in the spinal dorsal horn (178) and increases glutamate release *via* an interaction with presynaptic NMDA receptors (430). Acute cytokine application also enhances both the frequency and amplitude of sEPSCs in unidentified lamina II neurons (175). These authors reported a reduction in the frequency and amplitude of sIPSCs. The differences between this work and ours may not only represent the different time course of cytokine activation as Kawasaki et al. used 600 pM IL-1β in their work whereas we used a somewhat lower concentration of 100pM and observed differential actions on excitatory vs. inhibitory neurons.

Further analysis of fractalkine—microglia—IL-1β signaling led Clark et al. (167) to propose the following sequence of events. Soluble fractalkine activates CX3CR1 on microglial cells leading to the rapid release of IL-1β. IL-1β activates IL-1r on lamina 1 neurons and modulates function of postsynaptic NMDA receptors such that Ca^2+^ influx is increased when they are activated by glutamate. Elevated levels of intracellular Ca^2+^ in lamina I neurons activates phospholipase A2 leading to the liberation of arachidonic acid and the generation of prostaglandins. Within a few minutes of fractalkine application, prostaglandins increase transmitter release from primary afferents both directly and indirectly *via* iNOS activation and release of NO from microglia.

Presynaptic NMDA receptors have also been implicated in spinal actions of IL-1β where signaling between IL-1r and NMDA may be affected by the sphingomyelinase/ceramide signaling pathway to enhance glutamate release from the primary afferents in neuropathic rats (395, 430). IL-1β enhances endocytosis of glial glutamate transporters in the dorsal horn astrocytes through activating protein kinase C (431), the resultant augmentation of glutamate responses represents a complementary mechanism where cytokine enhances excitatory synaptic transmission.

## Actions of the Tertiary Mediator TNF-α in the Dorsal Horn

Acute activation of TNF receptor 1 by TNF-α inhibits the excitability of a subset of spinal GABAergic neurons. This effect involves p38 mitogen-activated protein kinase dependent suppression hyperpolarization-activated cation current (I_h_) (182). These effects have been reported to diminish with time suggesting TNF-α may be primarily involved with the induction rather than the persistence of neuropathic pain (40).

Although fractalkine action on microglia and potentiation of synaptic transmission in the dorsal horn involves IL-1β but not TNF-α (167), it does appear to facilitate long term potentaition (183). This has led to the suggestion that the differential contributions of TNF-α and IL-1β to fractalkine-induced enhancement of synaptic transmission may reflect the well-characterized phenotypic diversity of microglia (432). Thus, activation of microglia by different secondary mediators may result in release of specific mixtures of tertiary mediators which in turn promote diverse effects on synaptic transmission (183).

## General Comments Regarding Injury-Induced Signaling in the Spinal Dorsal Horn

### Role of Astrocytes; Initiation and Maintenance of Neuropathic Pain

Astrocytes become rapidly and persistently activated after peripheral nerve injury, suggesting they play a role in both the onset and maintenance of central sensitization (3, 433–435). As mentioned above, recent evidence also implicates microglial function in the long-term maintenance of neuropathic pain in animal models (377) but this may not be the case in all types of neuropathic pain in the clinic (3).

It is well-established that IL-1β from microglia stimulates astrocytic production of TNF–α and IL-6 as well as IL-1β itself (381, 434) thereby amplifying the initial IL-1β signal. Microglial derived IL-1β reduces the capacity of astrocytes to take up glutamate (179, 430) as a result of internalization of the astrocytic glutamate transporter (EAAT2) (179). Loss of EAAT2 function induces hyperalgesia, augmentation of glutamatergic synaptic responses and increased sensitivity of dorsal horn neurons to primary afferent stimulation (436, 437). Activated astrocytes have also been reported to release the NMDA receptor co-agonist D-serine (438) thereby augmenting overall dorsal horn excitability. Evidence for astrocyte involvement in the clinic has been obtained by post-mortem studies of HIV-patients with painful neuropathy (3). These authors showed that expression levels of the microglial markers CD11b and Iba1 were not elevated whereas the astrocytic markers GFAP and S100 beta were clearly increased. This was accompanied by increased levels of TNF-α and IL-1β, as well as components of MAPK signaling pathway, including pERK, pCREB, and c-Fos.

Since astrocytes are not the primary focus of this review, readers are directed to the recent review by Ji et al. (435) which underlines the role of astrocytic gap junctions and astrocyte derived chemokines in pathological pain. Several other comprehensive reviews have appeared (439–441) and recent work has underlined the role of astrocyte derived IL-17 in paxlitaxel induced pain (21).

### Ubiquitous Nature of Mediator Release and Effect

We have used the term primary mediator to cover substances released from the site of nerve injury, secondary mediator to describe substances released from primary afferent terminals and tertiary mediators to define substances released from microglia (Figure 1). Whereas, BDNF selectively released from microglia can be described as a tertiary mediator, production and effect of cytokines and chemokines is far more widespread. For example, IL-1β which is a classical macrophage derived signal, can be released from Schwann cells, microglia, astrocytes, neutrophils, granulocytes, mast cells and endothelial cells (2, 190, 381, 434, 442, 443) it would thus be classified both as a primary and tertiary messenger. In general it can be said that cytokines such as IL-1β can be released from more or less any cell type in response to an appropriate stimulus. IL-17 appears to be a primary mediator which is also released from spinal astrocytes in a model of chemotherapy pain (21).

Opening of the blood brain barrier is a well-known correlate of nerve injury induced allodynia (50, 444) and this may be initiated by aberrant afferent nerve activity (445). This enables lymphocyte and macrophage invasion of neural tissue. In addition, mediators generated in damaged nerves, microglia, Schwann cells or astrocytes might be expected to enter the circulation and exert actions throughout the body. This is supported by the observation that plasma levels of IL-1β are elevated in rodents subjected to spared nerve injury (446) or exposure to paclitaxel which models chemotherapy pain (52).

Mediators generated in the spinal cord would also be expected to have access to other brain regions *via* the CSF. IL-1β levels are increased in the CSF of patients with complex regional pain syndrome (275) and with thoracic disc herniation (447). Inflammatory mediators may also be elevated in the CSF of osteoarthritis patients (15).

Taken together these finding suggest that the diffusion of spinally and DRG generated mediators may gain access to other brain regions *via* both the CSF and systemic circulation. This may lead to mirror image pain following unilateral nerve injury (448) and/or mediator actions in higher brain regions that contribute to the analysis of nociceptive phenomena, the affective components of pain, sickness syndrome and formation of memory traces (446, 449). For example, microglia activation and BDNF release in the mesolimbic reward circuitry may contribute to the negative affect associated with chronic pain (185). With the possible exception of BDNF, all of the mediators described (cytokines, chemokines and Wnt ligands) can be released from multiple cell types and as such may play a role in the initiation or maintenance of neuropathic pain throughout the nervous system. Discussion of the actions of mediators in higher brain centers is outside the scope of this review.

### Sex

Neuropathic pain is seen more frequently in women than in men (29) and it is now recognized that understanding of divergent pain mechanisms in males vs. females is crucial to the development of appropriate therapeutic approaches (28, 33, 450, 451).

Investigations over the last 15 years or so have started to unravel cellular and molecular mechanisms that may contribute to this difference (29, 31–33, 452, 453). For example, microglia are not required for mechanical sensitivity to pain in female mice which require activation of adaptive immune cells such as T-lymphocytes (27, 320). The difference may result from a lack of P2X4 receptors in the microglia of females (26, 313). Despite this, behavioral responses to nerve injury in female rats are similar to those seen in males and both involve downregulation of KCC2 and perturbation of Cl- gradients (25). Because BDNF is not necessary for the development of allodynia in female rodents (27), the mediator released from adaptive immune cells remains to be determined. Similar findings have been found in the Freund's adjuvant *in vivo* model of inflammatory pain in rodents and confirmed in human neurons (33). These authors also showed that *ex vivo* BDNF enhanced synaptic NMDA receptor responses in lamina I neurons from males but not from females and that ovariectomy eliminated these differences. Importantly, the findings illustrate how sexual convergence onto shared cellular and behavioral endpoints, such as allodynia, pain sensitivity or KCC2 downregulation, may mask sex differences in underlying molecular and cellular mechanisms (28). Other recent work has shown that macrophage invasion of DRG is predominant in males and not in females although both show similar amounts of allodynia following peripheral nerve injury (454).

The realization that different mechanism are engaged to generate neuropathic pain in males vs. females has obvious therapeutic implications. For example, blockade of Na_v_1.8 channels in the peripheral nervous system with A-803467 is more effective in females than in males in a rodent model of joint neuropathic pain (455). Might blockers of Na_v_1.8 be more effective in women than in men? On the other hand, restoration of KCC2 function (456) may be effective in both males and females?

### Multiplicity of Signaling Processes

#### Different Injuries Different Mediators?

It is well-known that different types of nerve injury provoke different types or behavioral or physiological response. Thus, while mechanical allodynia produced by spared nerve injury persists for many weeks, that produced by chronic constriction injury is short-lived and recovery is seen in about 4 weeks (38, 142). Similarly, changes in synaptic transmission in the superficial dorsal horn are more robust after sciatic CCI than after complete sciatic nerve section (axotomy) (389). These findings may be consistent with an earlier observation that CCI promotes stronger and more long lasting upregulation of the primary mediators TNF-α, IL-1β, IL-10, MCP-1/CCL-2 in nerve stumps than nerve crush (306). Whilst neuropathic pain associated with multiple sclerosis is characterized by loss of spinal neurons (140), this effect is not produced with CCI (457, 458).

Recent work has shown how the nature of peripheral injury dictates the precise spinal circuitry involved in the generation of mechanical allodynia (459). Thus, neuropathic injuries generate allodynia by activation of excitatory protein kinase C gamma positive (PKCγ) neurons at the lamina II/III interface (460) whereas mechanical allodynia induced by inflammation involves excitatory calretinin positive neurons in inner lamina II (461). Cholecystokinin (CCK) positive neurons in laminae III-IV are important in both situations. Peirs et al. (459) also distinguished punctate allodynia (as produced by Von Frey filaments) from dynamic allodynia (produced by brushing a cotton swab across the hindpaw skin). This allowed them to identify a subset of CCK neurons which expressed the musculoaponeurotic fibrosarcoma oncogene homolog (Maf) and the transient vesicular glutamate transporter 3 (tVGLUT3), which are primarily involved in conveying dynamic rather than punctate allodynia.

Other work using knockout mice has shown that deficiency of CCL19/21 attenuates nerve injury evoked pain but not the hyperalgesia evoked by the autoimmune encephalomyelitis model of multiple sclerosis (149).

The above findings point to the possibility that different types of injury provoke the generation of different sets of mediators (276). This may be due to differential damage to various subsets of primary afferent fibers.

### A Paradox

The above sections outline the actions of many of the proposed primary, secondary and tertiary mediators involved in the development and persistence of neuropathic pain. There are several pathways by which a peripheral nerve injury can lead to pain but as shown in Tables 1–3, interruption of the actions of any single mediator seems to be capable of alleviating pain. For example, ATP activation of P2X7 receptors on microglia promotes release of IL-1β and activation of P2X receptors promotes release of BDNF. This would imply that it would be necessary to prevent the action of both IL-1β and BDNF to prevent the development of allodynia but it is known that inhibition of the actions of either individual mediator is effective. In other words if BDNF is inhibited why can't pain be initiated by IL-1β If IL-1β is inhibited why can't pain be initiated by BDNF? Also as mentioned above the actions of inflammatory mediators are mediated by a limited number of downstream signaling processes: ERK-MAPK signaling seems particularly important in this regard. If one signaling cytokine is blocked or knocked out why aren't its downstream effector mechanisms activated by other cytokines?

A better understanding of the interactions between mediators and their receptors and downstream effectors is clearly required for a more complete understanding of mechanisms underlying neuropathic pain in animal models that will lead to a better understanding of pain etiology in individual patients. This in turn may enable the application of personalized medicine approaches to pain management (459, 462).

## Author Contributions

All authors were involved in the writing and/or review of the manuscript.

## Conflict of Interest

The authors declare that the research was conducted in the absence of any commercial or financial relationships that could be construed as a potential conflict of interest.

## Publisher's Note

All claims expressed in this article are solely those of the authors and do not necessarily represent those of their affiliated organizations, or those of the publisher, the editors and the reviewers. Any product that may be evaluated in this article, or claim that may be made by its manufacturer, is not guaranteed or endorsed by the publisher.
